# A modular motion compensation pipeline for prospective respiratory motion correction of multi-nuclear MR spectroscopy

**DOI:** 10.1038/s41598-024-61403-w

**Published:** 2024-05-11

**Authors:** Stefan Wampl, Tito Körner, Martin Meyerspeer, Maxim Zaitsev, Marcos Wolf, Siegfried Trattnig, Michael Wolzt, Wolfgang Bogner, Albrecht Ingo Schmid

**Affiliations:** 1 High Field MR Center, Center for Medical Physics and Biomedical Engineering, Medical University of Vienna, Vienna, Austria; 2 Division of Medical Physics, Department of Diagnostic and Interventional Radiology, University Medical Center Freiburg, Freiburg, Germany; 3 High Field MR Center, Department of Biomedical Imaging and Image-Guided Therapy, Medical University of Vienna, Vienna, Austria; 4 Department of Clinical Pharmacology, Medical University of Vienna, Vienna, Austria

**Keywords:** Object tracking, Computer vision, Prospective, Retrospective, Motion, Motion correction, Motion compensation, Online, MR spectroscopy, Three-dimensional imaging, Magnetic resonance imaging, Molecular imaging

## Abstract

Magnetic resonance (MR) acquisitions of the torso are frequently affected by respiratory motion with detrimental effects on signal quality. The motion of organs inside the body is typically decoupled from surface motion and is best captured using rapid MR imaging (MRI). We propose a pipeline for prospective motion correction of the target organ using MR image navigators providing absolute motion estimates in millimeters. Our method is designed to feature multi-nuclear interleaving for non-proton MR acquisitions and to tolerate local transmit coils with inhomogeneous field and sensitivity distributions. OpenCV object tracking was introduced for rapid estimation of in-plane displacements in 2D MR images. A full three-dimensional translation vector was derived by combining displacements from slices of multiple and arbitrary orientations. The pipeline was implemented on 3 T and 7 T MR scanners and tested in phantoms and volunteers. Fast motion handling was achieved with low-resolution 2D MR image navigators and direct implementation of OpenCV into the MR scanner’s reconstruction pipeline. Motion-phantom measurements demonstrate high tracking precision and accuracy with minor processing latency. The feasibility of the pipeline for reliable in-vivo motion extraction was shown on heart and kidney data. Organ motion was manually assessed by independent operators to quantify tracking performance. Object tracking performed convincingly on 7774 navigator images from phantom scans and different organs in volunteers. In particular the kernelized correlation filter (KCF) achieved similar accuracy (74%) as scored from inter-operator comparison (82%) while processing at a rate of over 100 frames per second. We conclude that fast 2D MR navigator images and computer vision object tracking can be used for accurate and rapid prospective motion correction. This and the modular structure of the pipeline allows for the proposed method to be used in imaging of moving organs and in challenging applications like cardiac magnetic resonance spectroscopy (MRS) or magnetic resonance imaging (MRI) guided radiotherapy.

## Introduction

Magnetic resonance imaging (MRI), MR spectroscopy (MRS) and MR-guided radiation therapy in the thorax and abdominal region are notoriously affected by physiological motion. Investigating e.g. the heart, liver or kidneys requires strategies to deal with respiratory motion^1^. Many approaches have been employed, ranging from breath hold^2^, external sensors^3^ and MRI navigators in one^4^, two^5^ and three^6^ dimensions. Signals from external sensors like respiratory bellows^3^, cameras^7^ or pilot tone^8^ show variable correlation with internal organ motion. Gating methods using e.g. diaphragm navigators^4^ provide a reliable and robust way of dealing with motion. Yet, typically they prolong acquisition time considerably, rely on recurrent motion patterns and capture motion only qualitatively, i.e. without providing actual information about displacements in millimeters or degrees. By adopting patient-specific motion models^9^ or determination of individual calibration factors^10^, their performance can be substantially improved. However, exercise, stress tests, patient medication or poor compliance can heavily affect reproducibility and regularity of breathing patterns. In these conditions of irregular breathing patterns a direct and dynamic extraction of the organs’ displacement may be required and preferred to inflexible methods as e.g. gating.

Prospective motion correction allows to capture otherwise lost image information by following the moving target and measuring at an updated position. To do so, prospective methods have to directly apply motion information to update slice position^11,12^, brain MRS voxel position^13^ or radiation beam steering^14^. To achieve this, navigators and processing need to be very fast to minimize latency, reduce intra-navigator motion and minimize disruption of sequence timing. During radiotherapy, temporal resolution for respiratory motion updates is strongly recommended to be better than 500 ms^14^, while acquisition during end-systole for ECG triggered cardiac MRS even requires less than 250 ms^15^.

User-independent object tracking is widely used to automatically follow a target frame by frame in camera footage or movies^16^. Here, we investigate its application to MRI for the purpose of tissue tracking. Given typical optical image sequences (color, high resolution, rich in features) used during development and benchmarking of tracking algorithms in the OpenCV library (https://opencv.org), it was not evident which of the available tracking algorithms, if any, would work best with MR navigator images. Our MRI navigators provide grayscale images at comparatively low resolution and with few edges or features. Our approach was to test these with all the available trackers to evaluate their performance experimentally, and potentially identify object tracking as a promising, yet in MRI overlooked, motion extraction modality. We used two references: i) randomized human operators’ readings (labeled OP1) and ii) the RealTITracker (RTIT) algorithm^17^, which is not an OpenCV object tracker nor available on the scanner itself.

Here, we present a navigation framework for MR motion handling with the following features:

- multi-nuclear capabilities: ^1^H navigators for any other nucleus application, e.g. ^1^H, ^13^C, ^31^P, ^23^Na;
- navigation with local transmit/receive coils with inhomogeneous sensitivity and RF fields;
- quick response using OpenCV’s object tracking;
- combination of independent displacement vectors into one 3D translation vector;
- transformation to target sequence’s coordinate system;
- prospective and retrospective motion handling;
- modular approach: navigators, target sequence and localization methods can be independently exchanged;
- minimal user interaction.

For validation, motion phantom measurements were performed in a controlled setup. We chose in-vivo ECG-triggered cardiac ^31^P MR spectroscopy during end-systole to demonstrate the full feature set outlined above in a practical and realistic application with stringent timing requirements. The navigation performance was evaluated using motion phantom and volunteer measurements.

## Results

**Figure 1:**
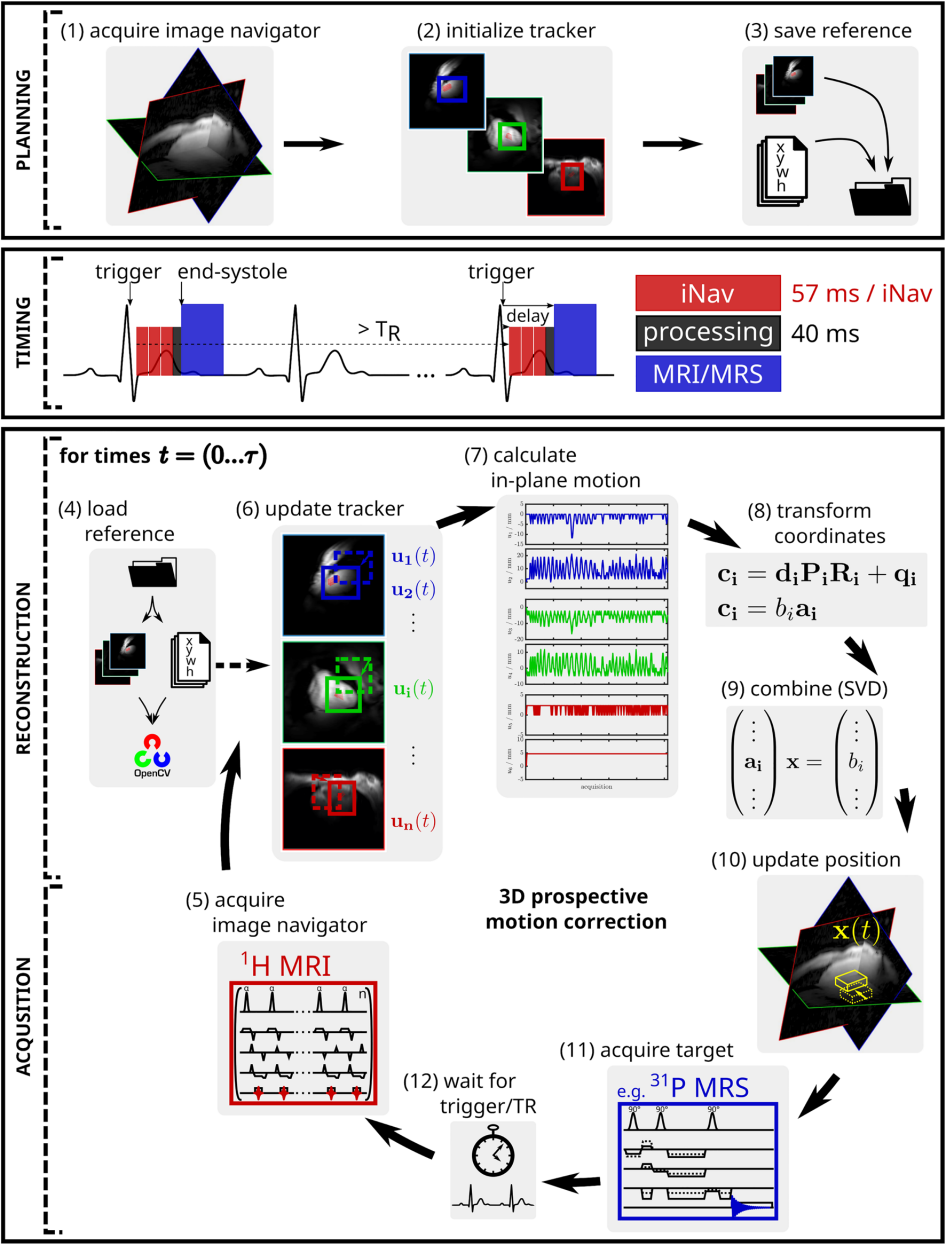
Pipeline depicting the extended motion compensation (*MoCo*) approach. During a separate planning scan (1), the region of interest on each image navigator is selected interactively by the user (2). These reference images and bounding-boxes are stored on the reconstruction system (3). Timing of the navigated, interleaved MR sequence with the image navigator (red), tracking and data processing (black) and the target sequence, e.g. a ^31^P MRS sequence (blue). The navigator may consist of an arbitrary number of slices (three shown here). In this example, the trigger delay before the navigators is set for the ^31^P MRS acquisition to occur during end-systole, which is typically 250–300 ms after the R-wave. With the start of the navigated MRS sequence, the references are loaded to initialize an independent OpenCV tracker for each image navigator (4). Following a trigger signal, the image navigators are acquired (5), reconstructed and the respective trackers are updated (6). In-plane translations (7) are transformed to the patient coordinate system (8) and combined to a single 3D translation vector using SVD (9). The vector is transferred to the target pulse sequence, e.g. MRS, to update the volume of interest (10) before signal acquisition (11). The cycle is repeated for each MRS or MRI transient (12). Transmit and receive frequencies are switched between each image navigator and target acquisition (^1^H and e.g. ^31^P, respectively) using multi-nuclear interleaving.

We developed a pipeline that is flexible in terms of target and navigator sequences, allowing for a number of different motion inputs, including error signals or weights and extensive motion data logging. The pipeline’s main components, as shown in Fig. 1, comprise a planning stage (1)–(3), the image based navigator acquisition (5), image navigator processing (6)–(10), and target MRI/MRS acquisition (11). Organ-independent motion estimation from arbitrary MRI slices was achieved by introducing OpenCV object tracking. Further, multi-nuclear interleaving demonstrates prospective motion correction capabilities and its multi-nuclear capabilities on ^31^P MRS.

**Figure 2:**
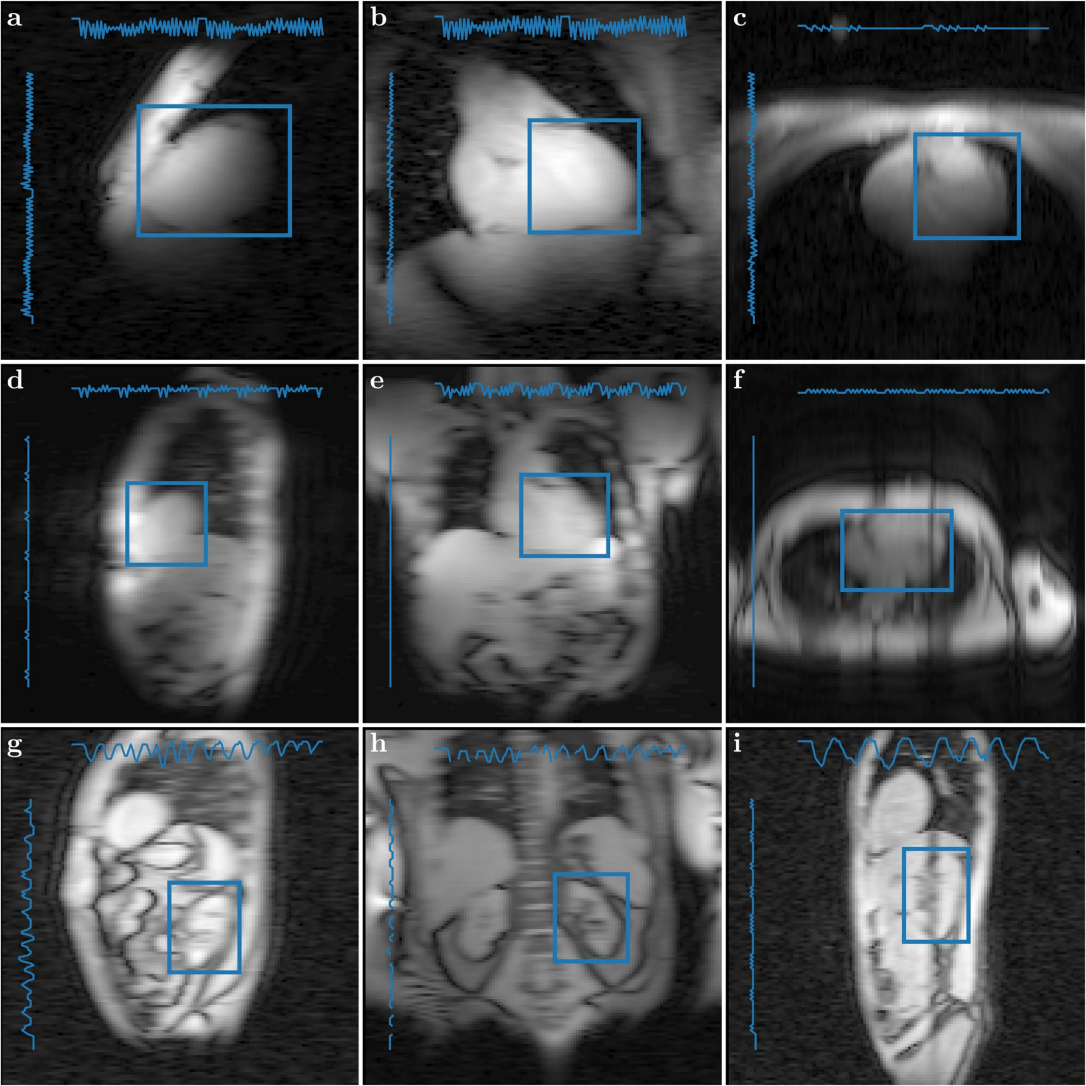
Various in-vivo MRI navigators demonstrating the wide range of possible appearances that can be handled by generic object tracking. The examples represent the heart (**a**–**f**) and kidney (**g**–**i**) at field strength 7 T (**a**–**c**) and 3 T (**d**–**i**) in sagittal (**a**,**d**,**g**,**i**), coronal (**b**,**e**,**h**) and transversal (**c**,**f**) orientation. Boxes mark the manually selected initial bounding boxes for object tracking. Lineplots indicate the motion amplitude of the whole time series to scale of the image resolution in the respective direction. A video of these navigators showing successful organ tracking with the kernelized correlation filter (KCF) algorithm can be found in the Supplementary Materials Videos S1 and S2. Detailed MRI parameters are specified in Suppl. Table S1 in the Supplementary Material.

**Figure 3:**
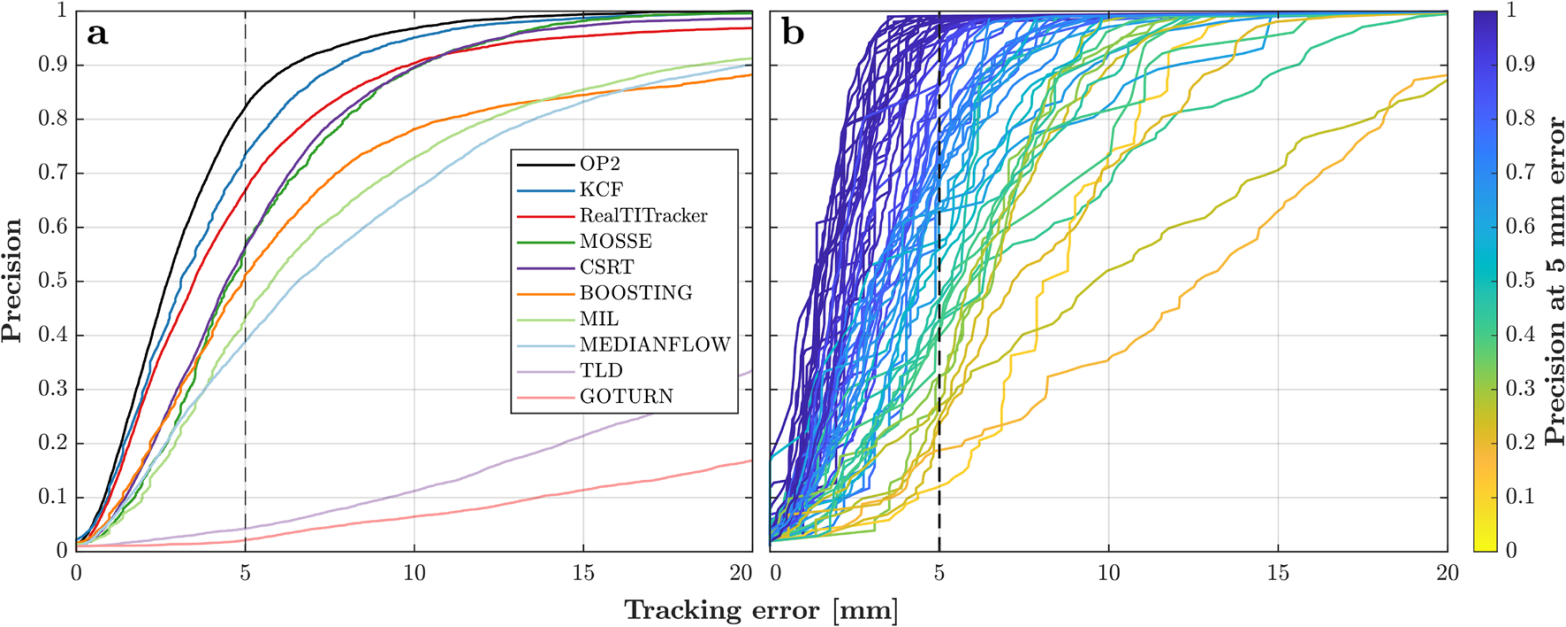
Cumulative density plot (‘precision curve’) of the tracking error. *Precision* is the fraction of all datasets for which tracking was better than a certain tracking error with respect to manual selection (OP1). The curves in (**a**) show the accumulated tracking errors of all 66 datasets for the automatic algorithms and the alternative manual selection (OP2). With a precision of 73.5%, KCF performs better than the RealTITracker (66.8%) and closest to the manual operator (OP2: 82.3%), while the other OpenCV trackers perform substantially worse. (**b**) shows only the KCF precision curves for all 66 datasets separately. The 5-mm threshold used for evaluation and comparison of *tracking precision* is indicated by the dashed line. Legend entries are sorted according these precision values.

The proposed method for prospective correction of MR acquisitions was successfully implemented into the manufacturer’s acquisition pipeline. The OpenCV library was directly linked to the reconstruction software avoiding potential delays due to network data transfer^18^. The feasibility was shown in phantom as well as in in-vivo experiments (Figs. 2, 3).

### Object tracking

Nine tracking algorithms were successfully applied to 66 navigator image series of volunteers and phantoms.

We found that in particular the KCF (Kernelized Correlation Filter) tracker performs very well on MRI data, even on images with low resolution and low contrast. A selection of navigator images is presented in Fig. 2, displaying the variety of image quality, both in contrast and resolution. Object tracking works on different organs (heart, kidney) on navigators in various orientations with a large variety of setups (3 T, 7 T, single loop coils, coil arrays) and imaging parameters (see Supplementary Materials Suppl. Table S1). The two Supplementary Videos S1 and S2 show organ tracking with the KCF algorithm on two sets of image navigator series. The boxes indicating the region of interest are following the respective organ, heart or kidney, in these cases.

**Figure 4:**
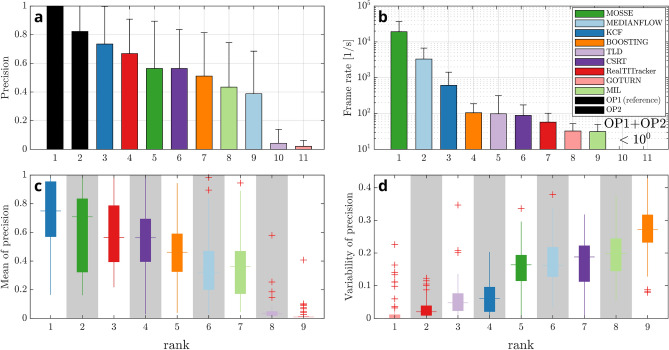
Ranking of the automatic trackers and the manual operators regarding the performance metrics precision (**a**), processing speed (**b**) and spatial robustness (**c**,**d**). (**a**) The precision at 5 mm tracking error compared to the randomized operator data (OP1), as indicated by the dashed line in Fig. 3a. Out of all automatic trackers, KCF performs best. (**b**) The trackers regarding their frame rate for an average tracking iteration. For evaluation of the spatial robustness, automatic tracking was repeated 125 times for each dataset with initial bounding boxes varying in size and position. The boxplots in (**c**) show the mean and in (**d**) the standard deviation of these sets of 125 precision curves at 5 mm tracking error. Regarding spatial robustness, the KCF tracker is clearly favorable with highest average precision and very low standard deviation. More detailed explanations on the spatial robustness evaluation can be found in the Supplementary Materials Suppl. Fig. S1a.

In total we performed the tracking task on 66 time-series of image navigators with 7774 images in total. Figure 3a shows a comparison of the analyzed trackers. The amount of iterations below a certain tracking error (here: 5 mm), *tracking precision*^19^, which is a commonly used metric to present the validity of the detected displacements, is used to evaluate tracking performance. The Euclidean distance between a reference’s and the tracker’s motion provides the tracking error, which in turn is sorted and displayed as a cumulative density plot (see Fig. 4). Two potential references were considered: results from manual selection (labeled OP1) or the RealTITracker (RTIT)^17^, which is not an OpenCV object tracker.

With a tracking precision of 73.5% KCF performs best of all trackers which, overall, is the closest to the manual operator (82.3%). With 66.8% the RealTITracker (Real-Time Image-based Tracker) ranks behind KCF (see Fig. 4a). The overall score, i.e. sum of the ranks in Fig. 4, is highest for the KCF (11), followed by MOSSE (13) and RealTITracker (16).

For evaluation of the spatial robustness, the tracking task was repeated 125 times with different initial bounding boxes each time, varying both in size and position. The average and the standard deviation of these 125 runs is presented in Fig. 4c,d, respectively. We found best spatial robustness for the KCF tracker with the highest average precision over all datasets of 74.4%. Hence, the average precision from runs with 125 different starting points is only marginally different from the single run reported above. For each dataset the standard deviation of the 125 precision curves was reported as their variability at the 5 mm error level (see Fig. 4d). The average variance over all 66 datasets was lowest for RealTITracker and KCF, which means that the size of the bounding box has minor influence on their tracking precision. The low variance of GOTURN (Generic Object Tracking Using Regression Networks) and TLD (Tracking-Learning-Detection) over the 125 tracking tasks is found in their vanishingly low mean precision, rendering them generally unsuitable for our application. For the KCF, only marginal differences between organs, between slice orientations and between field strengths were found (see Suppl. Fig. S1c in the Supplementary Materials for more details). Plots of tracker precision separately along phase and frequency encoding direction can be found in the Suppl. Fig. S2 of the Supplementary Materials. Most notably, the 3-4 fold higher resolution along frequency direction does only translate into a marginally better tracking performance. Also, the choice of reference, operator OP1, OP2 or RealTITracker, for calculation of the precision metric lead to similar results as presented above (see Suppl. Fig. S2 of the Supplementary Materials for more in-depth analysis).

Figure 4b ranks the average required processing time per frame for each tracking algorithm. MEDIANFLOW, a tracker based on median flow, and MOSSE (Minimum Output Sum or Squared Error) provided by far the fastest updates with update times below 1 ms per frame (Fig. 4), but their tracking precision is comparatively low (38.8% and 56.4%, respectively). KCF has the highest precision of all trackers and still achieves an update time of 2.6 ± 1.5 ms per frame. The optical flow method (RealTITracker) performs slowest (49.2 ± 7.8 ms) in our evaluation. Using the KCF tracker, less than 20 ms are needed for the whole navigator processing (including image reconstruction and coordinate transformations), meeting the requirements for a fast prospective position update.

Four of the available trackers internally report failure of tracking (KCF, MEDIANFLOW, MOSSE, TLD) if the confidence of the update is insufficient. Instead of performing a likely erroneous update, this allows for appropriate error handling and potential re-acquisition of the repetition. The reported failure rate of all tracked images (7774) was 0.1% (TLD), 1.5% (MEDIANFLOW), 6.1% (KCF) and 54.1% (MOSSE).

The overall score, i.e. sum of the ranks in Fig. 4, is highest for the KCF (11), followed by MOSSE (13) and RealTITracker (16).

### Validation of navigation in phantom

**Figure 5:**
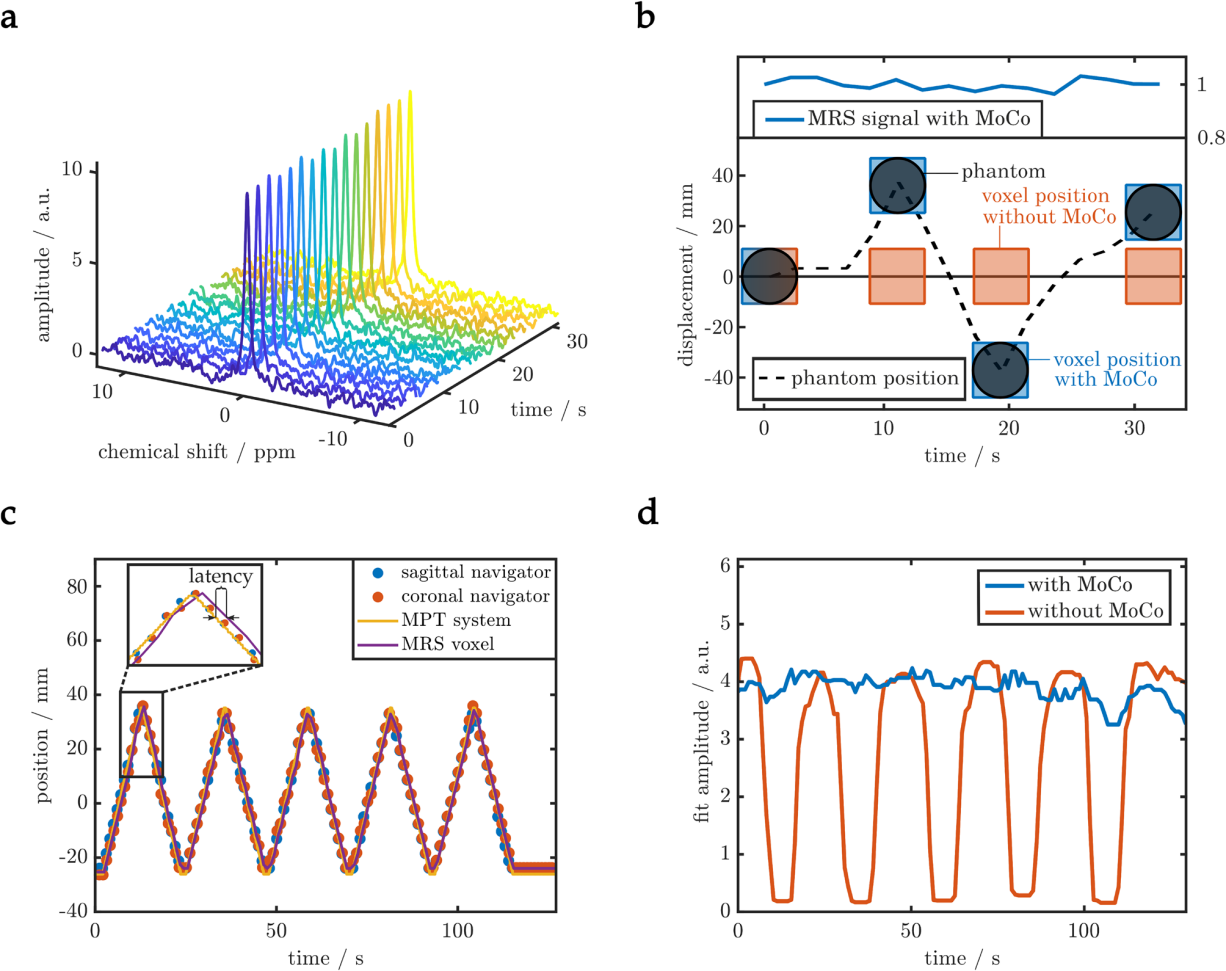
Validation of the motion compensation (*MoCo*) method in phantom scans. (**a**) The time series of ^31^P spectra acquired while the patient table was moved shows little fluctuations when the voxel position was updated prospectively using the proposed image-based navigation. (**b**) Illustration of the motion of the measured phantom (*d* = 20 mm), the voxel position along the z-axis with (blue) and without (orange) motion compensation and the measured signal amplitude when using motion compensation. The signal amplitude is preserved even when the target (sphere) is moved entirely from its initial position. (**c**) Using the motion phantom^20^ and the external MPT motion tracking system^7^ to confirm the correct tracking, combination and position update of the *MoCo* method. Image acquisition ($2 \times$ 114 ms) and processing (< 40 ms) induced a latency of less than 300 ms, as visible from the two position traces in the inset. (**d**) The signal gain with *MoCo* (blue) compared to without *MoCo* (orange) is apparent in the spectral data of these time series.

**Figure 6:**
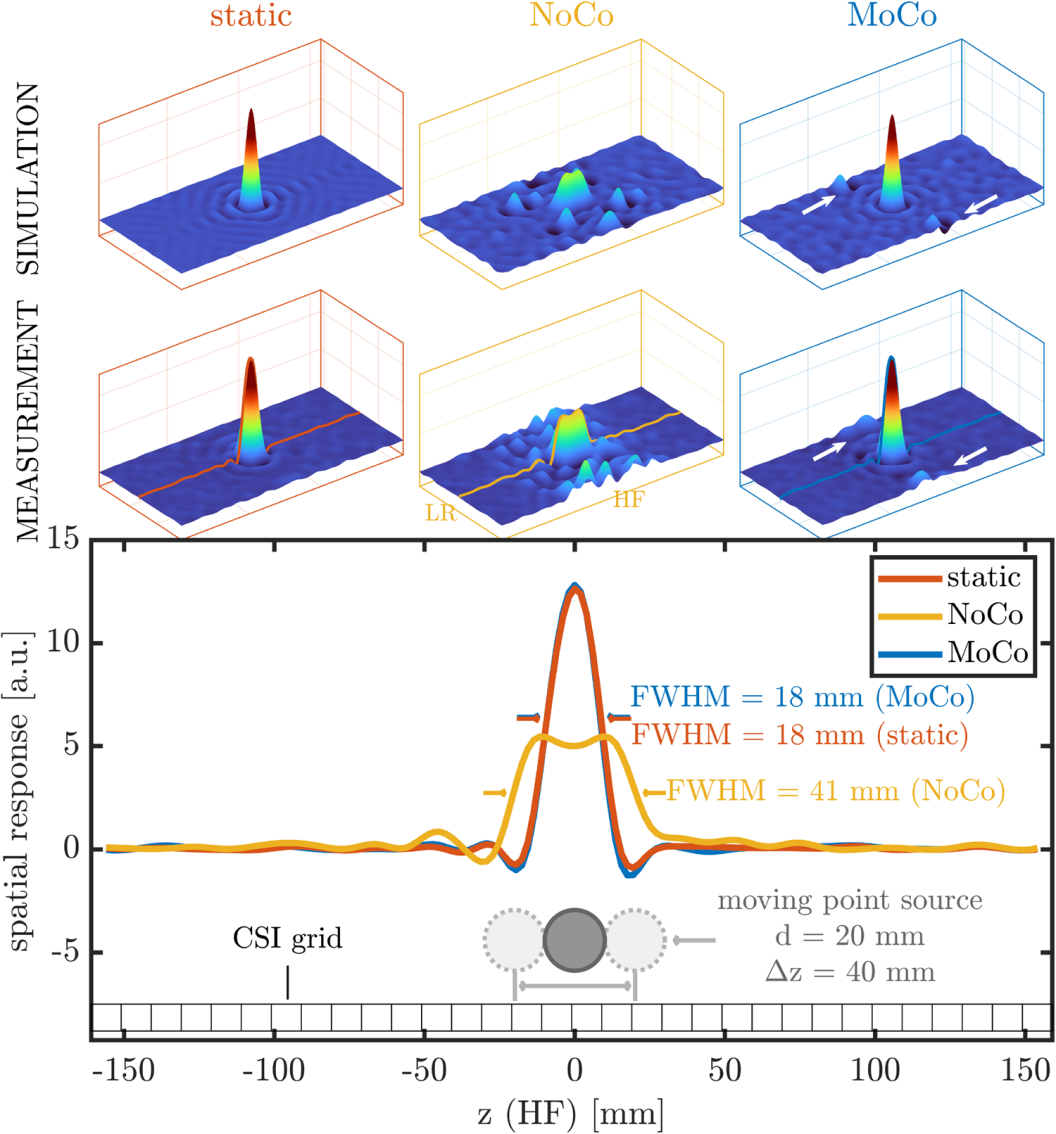
The effect of motion on the spatial response (PSF) of a point source using a 2D chemical shift imaging (CSI) sequence (matrix: 32 $\times$ 16, FoV: 320 $\times$ 160 mm, T_R_: 2 s, 1 average). The spherical phantom ($d = {20}$ mm) was filled with a concentrated phosphorus solution, moving periodically about the center position. Motion was step-wise by 4 mm per T_R_ along the head-foot (HF) direction with maximal displacement of $\Delta z = {40}$ mm. The first row displays the PSF derived from a simulation of the described phantom, motion and scan parameters. Plots in the second row show the real part of the actually measured and voxel-wise fitted spectral signal, spatially interpolated (5 $\times$) for better visualization. The compensated scan (*MoCo*) recovers the PSF of the static scan to a high degree. The non-compensated scan (*NoCo*) suffers substantial motional blurring and ghosting, visible from the signal loss in the center, a broadened PSF, articulate side lobes and signal bleed further from the center. Simulation and measurement are coherent. The remaining motion artifacts in the experimental data are also apparent in the simulation by incorporating the appropriate latency to the motion update (indicated by the white arrows).

The results of navigated MRS in a moving phantom is presented in Figs. 5 and 6. A navigator time series acquired from the phantom can be found in Suppl. Video S2 (i). The ^31^P spectra acquired from the small phantom show no decrease in signal amplitude (Fig. 5a,b) when prospective motion compensation (KCF) was applied. Note that the small phantom (20 mm diameter) was shifted by more than 30 mm which would have moved it entirely outside of the MRS voxel (20 $\times$ 20 $\times$ 20 mm), had it not been corrected for (Fig. 5b).

Accuracy of the motion tracking was validated using the MPT camera system^7^, see Fig. 5c. Shown are displacements of the two image navigators (sagittal and coronal) using the KCF tracker, their combination including processing latency (*MRS voxel*) and the external camera tracking data (*MPT system*). Mean absolute error (MAE) of the radial distances between *MPT system* and *MRS voxel* was 2.1 mm without latency and 2.9 mm when appreciating the latency of 295 ms, translating into a precision of 100% and 99%, respectively. Considering the pixel size of the navigators (2.7 mm isotropic), the tracking provides sub-pixel accuracy with MAE of 0.40 px, 0.37 px and 0.82 px in the three spatial directions sagittal, coronal and transversal, respectively. As expected, the MAE with latency along the main direction of motion (transversal) is largest (2.2 mm) but still in sub-pixel range (0.82 px). This confirms the correct combination of motion information gathered from multiple independent image navigators, plotted as *’MRS voxel’*. Without motion correction a substantial signal loss of 42% was found compared to the motion corrected measurement (Fig. 5d).

The impact of motion on the point spread function (PSF) of a chemical shift imaging (CSI) acquisition was assessed in simulation and experiment, as depicted in Fig. 6. Without motion compensation (*NoCo*) measurement data showed a significant deterioration of the PSF, due to various artifacts like motional blurring and ghosting. Simulation data provided conclusive results. In stark contrast, the compensated scan (*MoCo*) exhibited a well-preserved PSF with only minor remaining alterations. The simulations showed that these are mainly attributed to effects of processing latency, i.e. uncorrected motion occurring during the processing and updating stage of the sequence. These artifacts were pronounced along the first direction of phase encoding of the CSI sequence, which in this case was set to left-right (LR), orthogonal to the head-foot (HF) motion direction.

### Navigation in volunteers

We were able to extract useful motion traces from kidney and heart by object tracking using KCF, both with and without cardiac triggering. Supplementary Video S1 provides the whole time series of navigators presented in Fig. 2. Tracking was visually successful on liver (g–i) and triggered cardiac (a–f) acquisitions in all three major orientations (sagittal, coronal and transversal) using three different coils, a single and a dual loop surface coil, and a multi-channel flexible surface array. Saturation bands from the preceding navigator slice, visible in (f) and (i) do not impair tracking. Supplementary Video S1 presents examples for representative regular breathing patterns, while Suppl. Video S2 showcases several challenging navigator series. Tracking still performs well even in cases of irregular deep breathing (a+b), on acquisitions without cardiac triggering (c–g) in various scenarios from shallow to deep breathing, on series severely affected by B_1_ alterations (c,f,i) and through plane motion (b,g), with partial occlusion (c), torsion and contraction (h) of the target organ, on noisy (g) and high resolution (h) navigators alike, and for large displacements as in the phantom scan (i). Cardiac contraction modulates the motion curves notably without significantly distorting the tracking result.

**Figure 7:**
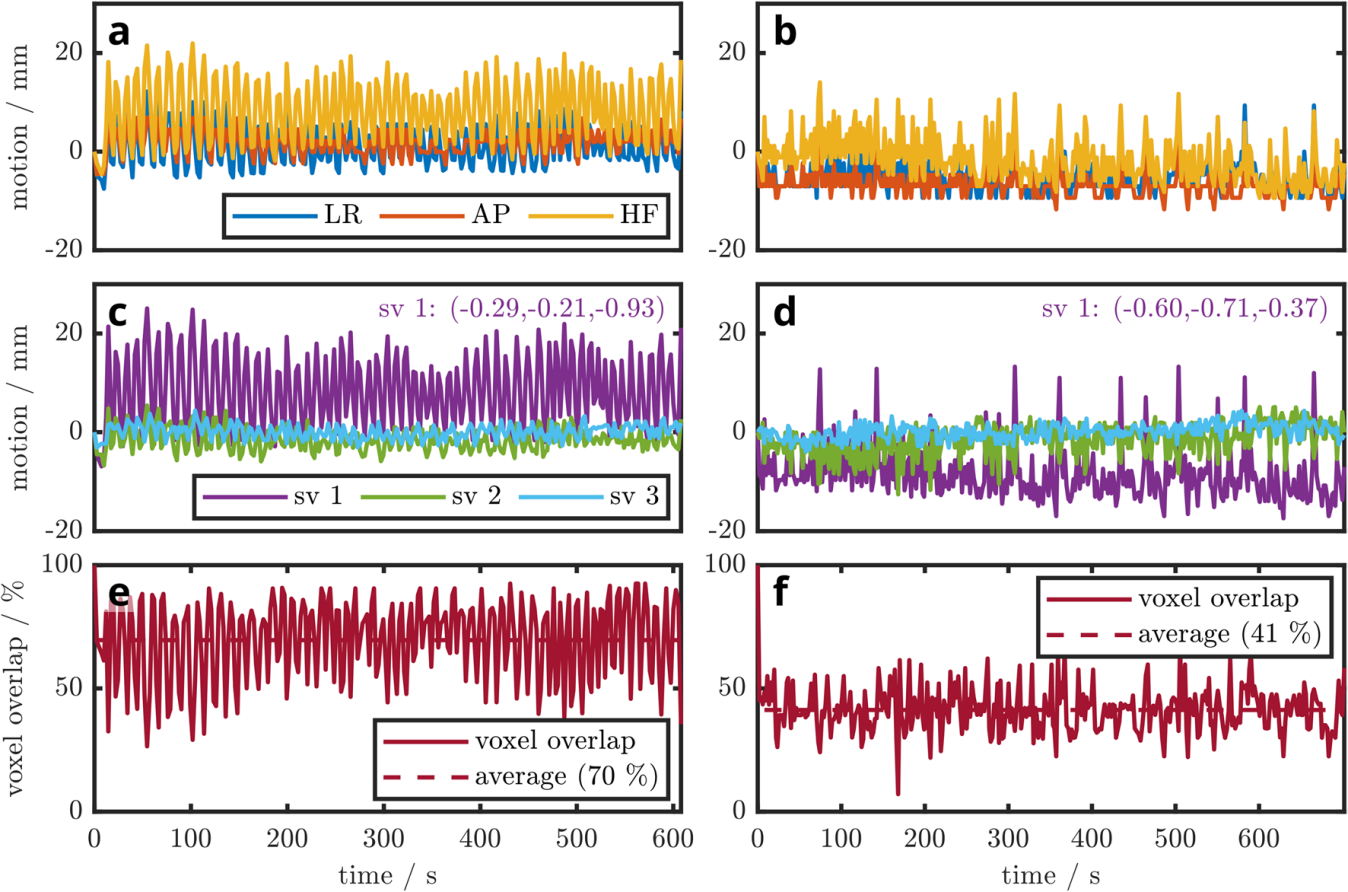
3D respiratory traces obtained from two volunteers by extracting and combining displacements from two orthogonal image navigators, respectively. Left: subject 1 with highly regular breathing pattern, recorded during the acquisition of spectra in Fig. 8a. Right: subject 2 with irregular breathing amplitudes and intricate patterns (according spectra: Fig. 8c,d). First row: motion curves in patient coordinates (**a**,**d**). Second row: curves decomposed into their principal directions (**b**,**e**). *‘sv 1’* denotes the individual principal component of the motion trace in patient coordinates (LR,AP,HF). Third row: to highlight the extent of voxel misalignment during motion, we demonstrate the overlap of the initial and the displaced magnetic resonance spectroscopy (MRS) voxel for single voxel spectroscopy (SVS) with 75 ml voxel size (**c**) and magnetic resonance spectroscopic imaging (MRSI) with 9.5 ml voxel size (**f**) in the third row. On average 70% and 41% of the nominal voxel volume coincides with the desired location over the whole measurement of $\approx$ 11 min. In (**f**) the position of the initial breath hold is never properly matched again, resulting in a poor yield of only 41% overlapping volumes.

The heart’s motion amplitude, estimated as the range from 10th to 90th percentile of a motion trace, were largest in sagittal (13.5 ± 9.9 mm) and smallest in transversal (3.9 ± 2.8 mm, Student’s t-test: $p<0.001$) directions. Accordingly, the main orientation of the cardiac displacement was found to be along the head-foot (HF) direction for most volunteers. However in more detail, the principal components of the 3D cardiac motion trajectories varied individually, ranging from heavily LR (LR: − 0.66; AP: − 0.60; HF: − 0.45) to almost exclusively HF (− 0.31; 0.00; − 0.95) in the most extreme cases. Figure 7 presents the motion traces of two volunteers projected on the three patient axes, as well as along the individual main motion orientations derived from singular value decomposition. The left column of Fig. 7 depicts the trace for a highly regular breathing pattern while the right column showcases irregular respiration amplitudes over the course of more than 10 min.

**Figure 8:**
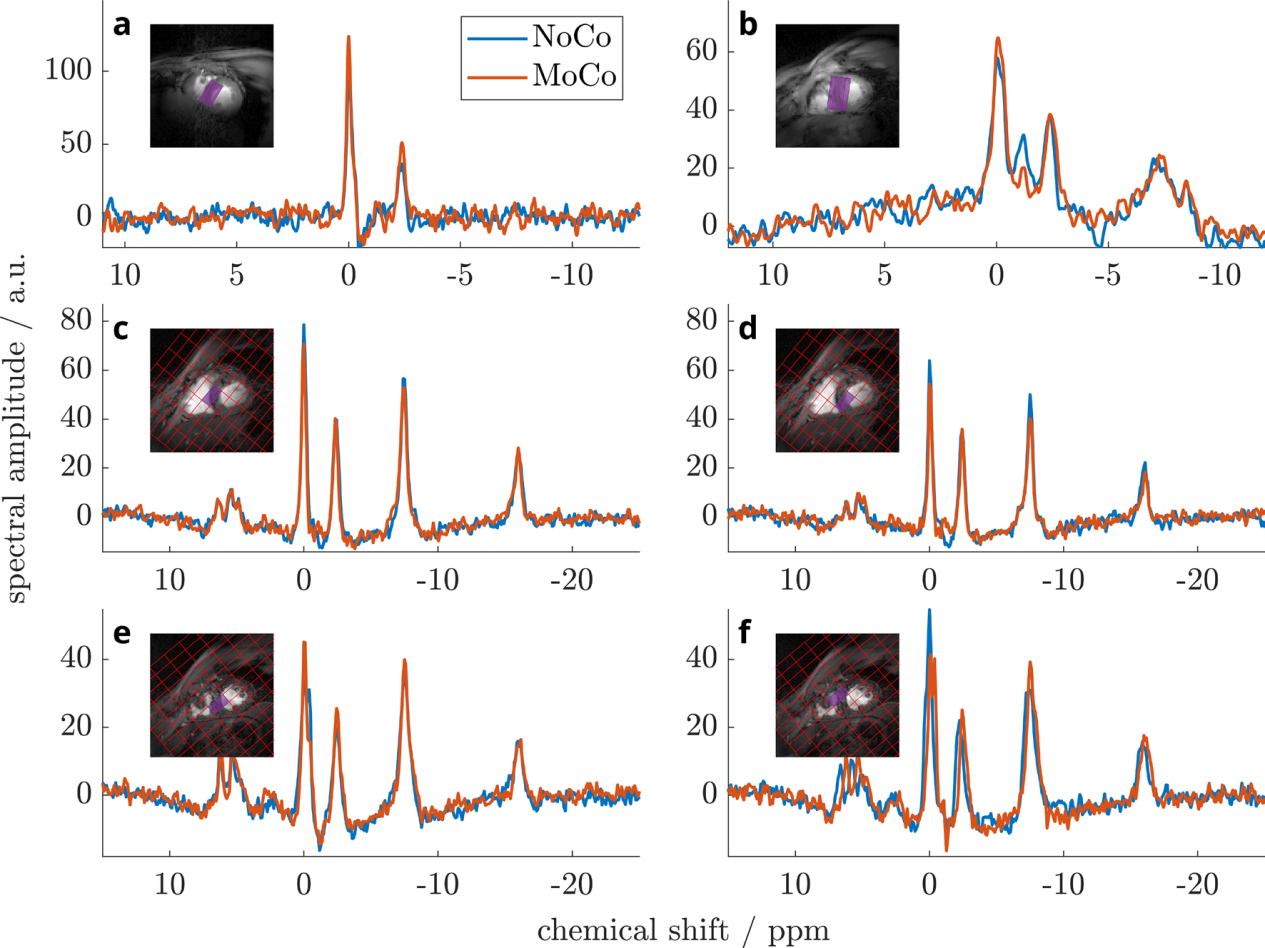
Exemplary in-vivo ^31^P spectra with and without prospective motion compensation using a stimulated echo acquisition mode (STEAM) sequence (**a**,**b**) and an acquisition-weighted chemical shift imaging (CSI) sequence (**c**–**f**) in four volunteers (#1: **a**, #2: **b**, #3: **c**+**d**, #4: **e**+**f**) at 7 T. (**a**) STEAM spectra with increased SNR in the *MoCo* case. (**b**) *MoCo* additionally reduces signal contamination between PCr and $\gamma$-ATP frequencies, most likely originating from skeletal muscle of the chest wall during breathing. (**c**,**d**) CSI acquisitions with *MoCo* result in lower PCr signal and reduced PCr/ATP due to less PCr contribution from skeletal muscle, while providing improved discrimination of P_i_ and phosphodiesters. Voxels close to the chest wall with localized shimming elucidate these improvements. (**e**,**f**) Contaminating PCr signals from skeletal muscle are distinctly reduced and can be more readily separated from cardiac PCr. Acquisition parameters for STEAM were: 75 ml voxel size, T_R_ = 3 s, T_M_ = 7.5 ms, T_E_ = 8.6 ms, 64 averages, triggering to end-systole; and for CSI: matrix 8 $\times$ 16 $\times$ 8, FoV 220 $\times$ 220 $\times$ 200 mm, 9.5 ml voxel size, T_R_ > 2 s, 3 averages, triggering to end-systole.

Prospective navigation was successfully executed in the available time window of 200–250 ms between physiological trigger signal and beginning of the MRS acquisition during end-systole (see “Timing” in Fig. 1). Exemplary in-vivo SVS and MRSI spectra of two healthy volunteers each are presented in Fig. 8, directly comparing *MoCo* and *NoCo* acquisitions. In the two subjects, motion corrected SVS spectra provide an increase in SNR (6 ± 4%), a decrease in line width ($-7 \pm 6$ Hz) and a notable reduction of contamination from skeletal muscle (Fig. 8a,b). Cardiac CSI spectra with *MoCo* show lower PCr/ATP in the septal voxels due to more accurate localization and less contamination from skeletal muscle (Fig. 8c–f).

## Discussion

We present a general framework for dealing with translational bulk motion in MRI and MRS. Several issues were addressed that had received rather limited attention so far, like navigators using local transmit coils and multi-nuclear applications, especially important at 7 T and beyond. We also demonstrated very fast navigation of several target tissues in under 250 ms while still providing full 3D translational motion estimates in millimeters. The pipeline provides prospective or retrospective motion correction of MRI or MRS sequences and is tailored but not limited to the particular requirements of high-field multi-nuclear applications. We show that OpenCV object tracking, especially the KCF tracker, provides robust image tracking on challenging, low-resolution MR image navigators without requiring any assumption or knowledge about the motion pattern. The flexible combination of information from multiple slices allows a full 3D translation. The feedback class of this pipeline allows it to incorporate any source of position or motion data, not just from tracking.

The navigator pipeline was developed using a framework for running a navigator sequence within a target pulse sequence, originally developed for brain MRS^21,22^. Both our navigator and target sequences are different, as well as the navigation part itself. However, we retained the parts which allow independent running of navigators from within a target sequence. We added multi-nuclear capabilities and encapsulated it into a shared library, which allows the navigator to be included into a variety of different target sequences with only the minimum of required adaptations. We present successful implementation on different scanners and software releases. The motion correction pipeline will therefore be directly portable to Siemens MR scanners of the VB and VE line, and with manageable effort to other scanners. Furthermore, the use of vendor-agnostic frameworks like Pulseq^23^ could be an option to use it on other scanners in the future. There are no restrictions for retrospective motion compensation, as object tracking can be run standalone on any image series or video.

Our implementation provides perpendicular motion information, offers flexibility with respect to choice of object tracking algorithm, and accelerates navigator acquisition. Most of the previous algorithms demand either high-resolution, homogeneous MRI sensitivity, reliable triggering, an extensive learning/training phase^24^, periodic motion patterns or they build on specialized, in-house built algorithms^25–27^. Object tracking, on the contrary, can deal with low quality data (low resolution and low contrast) and is comparably fast. Most algorithms do not require any previous knowledge or assumption about the motion amplitude, direction or periodicity, and can handle even disappearing displacement. Object tracking only requires the selection of the target region, a step implemented directly into the scanner software, requiring a single and easily manageable user interaction per patient.

We further present the capabilities in tracking accuracy without claiming superiority of the presented tracking algorithms. Comparison with the real-time image tracker (RealTITracker)^17^ shows the capabilities but also weaknesses of the best OpenCV tracker (KCF). KCF convinces with high speed and reporting of tracking failure, where RealTITracker scores with pixel-wise deformation-field output, an information available but largely discarded and compressed in our analysis. Tracking failure as reported by several trackers can be considered a useful feature as long as the failure rate remains reasonable. MOSSE for example reports more than every second repetition (54%) as erroneous, which will practically not be beneficial, despite its impeccable speed. A reasonable amount of failure (e.g. 6% for KCF) is however an advantage, allowing for re-acquisition with proper position update where other trackers would blindly report potentially erroneous tracking information. Overall, the KCF tracker performs closest to the second operator and best out of all automatic algorithms, regardless of the reference chosen, OP1, OP2 or RealTITracker. Further, KCF ranks highest regarding all described metrics: precision, processing time and the spatial robustness.

A novelty is that we linked the OpenCV object tracking library directly into the scanner’s reconstruction pipeline ICE (Siemens’ Image Calculation Environment). OpenCV is a powerful image-processing library and our pipeline allows full access to all its tracking functionality during image reconstruction and processing. We believe that this opens the door to a wide range of applications with less stringent requirements on navigator acquisition. Building on the actively maintained OpenCV toolbox, our motion compensation directly benefits from future computer vision developments integrated into OpenCV.

To facilitate setup, typically two or three perpendicular navigator slices, parallel to the anatomical body planes (sagittal, coronal or transversal), suffice to cover target motion in three dimensions. In our experience, sagittal and coronal slices are best suited for cardiac tracking, since transversal slices experience the highest degree of through plane motion and do not contribute substantial new information. However, the pipeline is not limited to these planes but permits the selection of any arbitrary orientation. In principal, this allows for navigator slices to be aligned to the main directions of organ motion to reduce through plane motion, at the cost of prolonged and elaborate setup. Based on several acquisitions (data not shown), the cost of this fine-tuning outweighs the benefit of marginally improved tracking performance. Additional parallel, perpendicular or oblique navigator slices can still be added to improve spatial coverage or tracking accuracy. Similar to acquiring more phase encoding lines or improving image resolution, this obviously increases delays for the target acquisition.

The variability in tracker performance between datasets is mostly linked to image resolution. Use of parallel imaging or other under-sampling techniques could be used to improve navigator speed or quality. This may result in increased tracking precision as long as time for image reconstruction is kept sufficiently short. Even with a single loop dual frequency coil (used for ^31^P MRS at 7 T), which does not permit any parallel imaging acceleration, navigation performance was remarkably well, given the short time window available for navigator acquisition and tracking calculations for cardiac MRS during end-systole.

The largest portion of the pipeline’s duration was spent on acquisition of navigators. In most cases we therefore chose two or three navigator slices with an acquisition time of 60–90 ms per slice to fit navigator acquisition (< 200 ms) and processing (< 40 ms) all within systole, i.e. between the cardiac trigger signal and end-systole. A shorter navigator acquisition would also reduce inter- and intra-navigator motion, contributing to a further reduced tracking error. With an overall time for navigation of less than 180 ms for a two-slice navigator (2 $\times$ 70 ms $+$ 40 ms), our approach shows a comparable performance to 218 ms for single slice MLC tracking in MRI-linac^28^, while additionally providing perpendicular motion information.

The heart can be challenging to image due to its deformation, the constant motion and the pulsating strong blood flow. However, for motion tracking the heart offers a rather easy target because of the relatively sharp contrast against its surrounding tissues, mainly air in the lungs. We also present equally reliable results from several datasets of kidney navigators to show the capability of object tracking with weaker contrast. The videos in the supplementary materials showcase several typical but also difficult tracking situations. Especially the successful tracking in Suppl. Video S2 shows that object tracking can handle a variety of challenging situations: improper navigator planning, insufficient navigator contrast, shallow, deep or irregular breathing, predominant thoracic or abdominal breathing, through plane motion, or B_1_ inhomogeneities of small surface coils. Also, object tracking performs well even without cardiac triggering, regardless of the timepoint in the cardiac cycle. Consequently, regularity of cardiac contractions, i.e. arrhythmia should not pose a substantial obstacle for the selected algorithms. These examples underline the usability of object tracking also in clinically relevant applications as exercise and stress tests, or in cases of arrhythmia or poor patient compliance.

We also present the extraction of individual breathing patterns from the navigator data (Fig. 7). This direct and internal information could serve as a calibration tool for external correction methods or gating approaches^10^, or serve as a basis for the decision of appropriate motion mitigation techniques.

Exhalation or inhalation during breath holds often exaggerate the extreme positions of free breathing. We found that the exhaled end-position during free breathing was often substantially less extreme than the exhaled breath hold position during the planning stage, e.g. for cardiac localizers. While our prospective approach intrinsically corrects for that, different approaches, as e.g. gating might suffer from frequently rejected acquisitions when using typical acceptance windows. However, larger windows to mitigate these rejections lead to larger voxel-to-target displacements. Figure 7 illustrates this very convincingly in the graphs of the bottom row, with potentially only 41% overlap of the voxel with its intended position in the tissue.

Non-proton MR acquisitions, as e.g. ^31^P, ^13^C or ^23^Na, are increasingly used to complement diagnosis and prognosis by conventional methods due to the unique insights they allow into tissue metabolism and viability^29–31^. Many non-proton MR pulse sequences to date still lack implementation of appropriate motion compensation methods, partly also because multi-nuclear interleaving may run on the MR scanner as is^32^ but often requires hardware modifications, e.g. for Siemens VB-line scanners^33^.

We demonstrate the feasibility of our prospective navigation approach to two different MRS localization schemes: single voxel spectroscopy (SVS) and magnetic resonance spectroscopic imaging (MRSI). We include proof of principle cardiac ^31^P MRS data obtained from two volunteers for each of the localization methods, using stimulated acquisition mode (STEAM) and chemical shift imaging (CSI), respectively (Fig. 8). Spectral quality of in-vivo acquisitions was improved slightly when applying motion compensation, a finding in line with previous reports by Kozerke et al.^34^. The effect on in-vivo ^31^P spectra may be small, but *MoCo* convincingly improves spatial accuracy, as can be deduced from Fig. 6. This supports the interpretation of our smaller PCr/ATP values as being a consequence of better localization, therefore less spectral contamination, and hence more accurate in-vivo values.

Acquiring navigator information in close temporal proximity to the Magnetic Resonance Spectroscopy (MRS) acquisition offers the possibility of correcting chemical shift imaging (CSI) data either retrospectively or prospectively. This correction approach facilitates acquisitions with minimal modifications to the original CSI protocol, thereby preserving achievable repetition times (T_R_) and, consequently, maintaining acceptable total acquisition duration. Additionally, this approach ensures optimal signal-to-noise ratios, a feat that is challenging to attain through alternative methods such as respiratory gating^35^.

Effects of motion on the point spread function (PSF) of CSI acquisitions were confirmed by a simulation of a motion corrupted k-space as described previously^35^. Considering uni-directional, periodic motion during coherent k-space sampling patterns (e.g. line by line), some very striking artifacts remain in the CSI data. Incorporation of the *MoCo* latency into the model explained the majority of the remaining artifacts in the *MoCo* acquisition. Interestingly, the relative orientation of motion and Cartesian encoding direction determine the nature of the artifacts appearing, while the broadening of the PSF is primarily dominated by the motion direction. Evidently, the application of motion compensation remedies the degradation of the PSF in a CSI acquisition, as presented in Fig. 6. This highlights the effectiveness of motion compensation in mitigating the detrimental impacts of motion.

Our method currently handles 3D motion based on the information of 2D images. Among others, the reasons for this are acquisition speed and resolution. This, in turn, leads to potential difficulties when faced with through-plane motion. While several different slice orientations mitigate the problem considerably, false apparent in-plane motion could be detected if shape and location vary considerably from plane to plane. A good choice of both navigator orientations and tracking regions can usually address this.

Another limitation in the current implementation is that we consider only rigid body translations. This is definitely a simplification of what is happening, e.g. during breathing motion. Ultimately, pixel-wise deformation generation as by the RealTITracker^17^ provides the most complete picture of the organ motion. For application in MR spectroscopy, this information eventually has to be condensed into six degrees of freedom (3 translations, 3 rotations). Since the navigator slices, the tracking volume and the relatively large MRS voxels are all placed on top of the region of interest, translations are the most prominent motion component, at least for the heart. With smaller voxel sizes, as e.g. in ^1^H MRS, the relevance of rotations might however be raised. The pipeline should be extended to handle rotations or non-linear transformations as well as multi-target tracking. The feedback class is an excellent starting point for this since it allows for a seamless introduction of a motion field. For example multiple trackers from different areas on the same slice could provide such data, while not substantially prolonging the navigator block.

Further, we currently do not compensate for neither B_0_ nor B_1_ field alterations. These may lead to serious changes in signal amplitudes, intravoxel dephasing, blurring or line-broadening of the target signal. Processing the MRS signals separately, based on the current motion state, before reconstruction may mitigate some but not all of the above. Dynamic shimming reportedly improves spatially (respiration) and temporally (cardiac cycle) induced B_0_ alterations^36^. This requires further implementation steps, in combination with additional setup time and acquisition delays^37^. However, the moderate changes of B_0_ are outweighed by more substantial B_1_ alterations, which could be optimized by special RF pulse design^38^.

Adapting more target sequences, especially for (multi-nuclear) imaging, would be the next step. It is intended to implement and test our navigation pipeline for other modalities, in particular ^1^H MRS and cardiac MRI. We think, that this would also be a useful tool in MRI-guided radiotherapy, fetal or pre-clinical imaging.

A seamless access to OpenCV could stimulate further development in image reconstruction and post-processing and its translation to clinical scanners.

## Conclusion

We describe a flexible framework to be used in torso MRI, especially in challenging applications involving multi-nuclear imaging, higher fields and the heart. Computer vision object tracking is a highly versatile tool that works out-of-the-box, reasonably fast and with high accuracy even on low resolution MR images.

## Methods

The pipeline was partially based on a previously presented framework developed for brain MRS^21,22^. We retained the parts which allows it to run independent navigators from within a target sequence. Heavy modifications were required, i.e. the navigator sequence, the way motion information is obtained and combined, as well as to accommodate the needs of multi-nuclear acquisitions, 7 T scanners and target tissues, i.e. heart and kidney. The navigator was bundled in a shared library to be included into practically all sequences with only minor adaptations.

### Data acquisition

For all measurements, Siemens MR scanners (3 T Magnetom Prisma^Fit^ and 7 T Magnetom before and after Dot+ upgrade) of software releases VB17, VE11C, and VE12U were used. During the in-vivo measurements several surface (array) coils, partially with multi-nuclear capabilities, were tested for the use with our pipeline. At 3 T data acquired with the manufacturer’s 32-channel spine coil *Spine 32*, the manufacturer’s 18-channel flexible surface array *Body 18* (Siemens, Erlangen, Germany) and a dual-tuned phased-array 2-channel ^1^H/8-channel ^31^P coil (Stark Contrast MRI Coils Research, Erlangen, Germany) are presented (coils “1”, “2” and “3”), respectively, in the Supplementary Materials Suppl. Table S1. At 7 T a single-loop dual-tuned ^1^H/^31^P-coil (14 $\times$ 22 cm ^1^H, 14 cm ^31^P, Rapid Biomedical, Rimpar, Germany) was used for phantom and in-vivo acquisitions. Further two dual-tuned surface coils were employed for single phantom measurements: a single-channel ^1^H/^31^P-coil with 10.5 cm ^1^H and 9.5 cm ^31^P channels (Rapid Biomedical, Rimpar, Germany), and a two ^1^H/three ^31^P channel coil anatomically fitted to the human calf muscle (^1^H: 17 $\times$ 12.5 cm, ^31^P: 15 $\times$ 10 cm)^39^.

The study was approved by the ethics committee of the Medical University of Vienna, Austria, and conducted according to the Declaration of Helsinki in its latest version. Informed consent was obtained from all participants. Sixteen healthy subjects (6f/10m, age 22–47 years, body mass index 16.8–24.9 kg/m^2^) were studied in supine position. Further measurement details are summarized in the Supplementary Materials Suppl. Table S1.

### Navigator module

Image quality of the navigators was deliberately sacrificed in favor of acquisition speed. The MR image navigator was a fast-low-angle-shot (FLASH) pulse sequence (RF pulse duration: 600 µs, flip angle = 12^∘^, receiver bandwidth: 1500 Hz/px) with short T_E_ = 1.03–1.19 ms and T_R_= 2.2–3.6 ms) with a reduced set of phase encoding lines without RF spoiling. Acquisition parameters are reported in full detail in the Supplementary Materials Suppl. Table S1. To fit specific requirements, all imaging parameters, as e.g. resolution, contrast, slice orientation or field of view, have to be set before navigated acquisitions in the so-called “planning scan” (step 1 in Fig. 1). There are no limitations to neither the quantity, the orientation, nor the position of the selected navigator slices, but they can be selected freely as required.

### Motion extraction module

To extract displacement information from the 2D image navigators we opted for object tracking provided by the open-source computer vision library OpenCV (https://opencv.org). This has several advantages:

1. algorithms are highly generic and not limited to a specific tissue, motion pattern or image resolution,
2. high processing speed,
3. availability of multiple object tracking algorithms packaged in one toolbox,
4. ongoing development by an active computer vision community,
5. relatively simple updating to newer OpenCV versions.

The object tracking was directly linked into the scanner’s reconstruction pipeline ICE (Siemens’ Image Calculation Environment). Development was primarily done in C$++$ on Windows and Linux, using OpenCV v3.2. Object tracking requires the manual selection of a bounding box on a reference image (step 2 in Fig. 1). This initialization is realized on the planning scan directly from the MR scanner’s user interface “Syngo”, which integrates seamlessly into the measurement procedure. Initialization images and bounding box data were stored for later reference (step 3 in Fig. 1) and used during the update process (step 4 in Fig. 1). The object tracking of this framework was also implemented as a post-processing tool directly accessible from the Syngo menu. It can be performed on any single- or multi-slice time-series of images.

#### Tracking algorithms

The OpenCV tracking API contains implementations for several tracker algorithms, all accessible via a single interface. A brief overview of the methods shall be provided here, for a general review see also reference^40^. The BOOSTING algorithm uses a discriminative appearance model that is trained online on one positive example, defined by the initial bounding box (online boosting), to separate the object from the background^41^. For a new frame, evaluation of the model on multiple neighboring pixels highlights the most probable location by its maximal score. The classifier is updated with the positive example with each new frame, however, it lacks reliable reporting of tracking failure. The multiple-instance learning (MIL) tracker extends this idea by considering a whole bag of potentially positive image patches through multiple instance learning. This improves flexibility and performance during significant appearance changes or partial occlusion^42^. As the patches of the MIL tracker govern multiple overlapping pixels, these redundant data can be leveraged to reduce processing time by orders of magnitude. With the kernelized correlation filter (KCF) tracker, Henriques et al. demonstrated that the resultant data and kernel matrices can be represented by circulant matrices which leads to mathematically advantageous properties in the Fourier domain^43^. These allow the KCF tracker to incorporate a much larger number of negatives at a substantially higher processing speed. Counter-intuitively this makes it faster and more robust at the same time. The tracking-learning-detection (TLD) tracker decomposes its task into the components tracking, learning and detection which results in better performance under large motion, full occlusion and substantial target scaling^44^. MEDIANFLOW follows the object in forward as well as in backward direction, calculates the error between the two trajectories and consequently reports reliable tracking failure^45^. Minimum Output Sum of Squared Error (MOSSE) uses adaptive correlation filters with robust results under scaling, pose changes and non-rigid deformations^46^. GOTURN (Generic Object Tracking Using Regression Networks) is a tracker based on a pre-trained Convolutional Neural Network (CNN) and therefore provides very fast processing^47^. The CSRT (Channel and Spatial Reliability Tracking) tracker is based on a discriminative correlation filter with channel and spatial reliability^48^. Usage of a spatial reliability map constrains the tracker stronger to the actual object, resulting in improved tracking of non-rectangular objects.

### Motion compensation module

The motion compensation module creates the interface between online reconstruction (Siemens’ Image Calculation Environment - ICE), tracking (OpenCV) and the pulse sequence runtime environment. In this newly developed implementation, we introduced a feedback class that holds an arbitrary number of position features from different slices and potentially even from non-MRI sources, e.g. external sensors. The delay for the navigator image reconstruction, the update of the tracker and the transmission of the translation information typically did not exceed 40 ms.

In the following, we describe the generation of all features, applying the respective coordinate transformation and combining the features into a single 3D translation vector in more detail. This procedure is performed anew for each MRS repetition, i.e. once every T_R_, when an update of the voxel position is necessary.

#### Features

We denote features by index $i=1:m$, with *m* being the total number of features per repetition. The spatial displacement relative to the reference scan is calculated for each feature individually and then transformed to the common patient coordinate system (steps 7+8 in Fig. 1). A full feature $\mathbf {f_i}$ is composed of three components:

1. Its direction in form of a 3D unit vector $\mathbf {a_i} = (a_{ix},a_{iy},a_{iz})^\textsf{T}$ in the patient coordinate system,
2. Its respective length $b_i$, e.g. the displacement calculated from tracking, and,
3. (optionally) A weight $w_i$ to potentially emphasize features with higher confidence over others.

Any motion information, also from external sensors, can contribute to the position update as a feature $\mathbf {f_i}: \{\mathbf {a_i},b_i,w_i\}$. In our case, each of the *k* navigator slices contributed four features, one for each edge of the object tracking bounding box, resulting in $m=k \times 4$ features. While this delivers redundant information in the case of rigid bounding boxes, it provides the flexibility to also handle bounding boxes with changing dimensions. All features *i* were treated equally with $w_i = 1$.

#### Coordinate transformation

In general, any in-plane vector $\mathbf {d_i}=(d_{ix},d_{iy})^\textsf{T}$ can be transformed from its 2D image coordinates to a common 3D coordinate system, as e.g. the patient coordinates, by a sequence of rotations and translations:

1 $$\begin{aligned} b_i \mathbf {a_i} = \mathbf {R_i} \mathbf {P_i} \mathbf {d_i} + \mathbf {q_i} \end{aligned}$$

The diagonal matrix $\mathbf {P_i}=\textrm{diag}\{p_{ix},p_{iy}\}$ scales the vector by the pixel size. The matrix $\mathbf {R_i}=(\mathbf {e_{i1}}, \mathbf {e_{i2}})$, filled by the two 3D unit vectors spanning the image plane, projects the vector into the 3D patient coordinate system. And, $\textbf{q}=(q_x,q_y,q_z)^\textsf{T}$ applies the shift from the scanner origin to the slice origin.

#### SVD combination

The combination of all $i=1:m$ available features $\mathbf {f_i}$ can be written as a system of linear equations:

2 $$\begin{aligned} {\tilde{{\textbf{A}}}}\textbf{x} = \textbf{W}\textbf{A}\textbf{x} = \textbf{W}\textbf{b} = {\tilde{\textbf{b}}} \end{aligned}$$

Here, the weights $w_i$ are applied in the form of a $m \times m$ diagonal matrix $\textbf{W}$, $\textbf{A}$ is a $m \times 3$ matrix holding the directions $\mathbf {a_i}$ of the *m* features, and $\textbf{b}$ is a column vector of the *m* feature lengths $b_i$.

Singular value decomposition (SVD) delivers a low rank approximation for the rectangular matrix ${\tilde{{\textbf{A}}}}$ via the factorization

3 $$\begin{aligned} {\tilde{{\textbf{A}}}} = \textbf{U}\textbf{S}\textbf{V}^\textsf{T} \end{aligned}$$

with the singular values $\sigma _i$ in the diagonal matrix $\textbf{S}$^49^. The columns of the matrices $\textbf{U}$ and $\textbf{V}$ are composed by the left singular vectors $\mathbf {u_i}$ and right singular vectors $\mathbf {v_i}$, respectively. The best approximation for the 3D translation vector $\textbf{x}$ is consequently found as the three highest singular values of $\textbf{A}$:

4 $$\begin{aligned} \textbf{x} = \sum _{i=1}^{3}\frac{\mathbf {u_i}^\textsf{T}\textbf{b}\mathbf {v_i}}{\sigma _i}. \end{aligned}$$

Regularization of the singularities in $\sigma _i$ was obtained by substituting $\frac{1}{\sigma _i^2} \rightarrow \frac{\sigma _i^2}{(\sigma _i^2+\tau ^2)^2}$ with $\tau = 0.05$. The resulting 3D translation vector $\textbf{x}$ in patient coordinates (step 9 in Fig. 1) is then forwarded to the target pulse sequence for position update.

### Target sequence module

The framework was incorporated into two established magnetic resonance spectroscopy (MRS) pulse sequences, a single voxel spectroscopy (SVS) stimulated echo acquisition mode (STEAM) sequence^50^ and a MR spectroscopic imaging (MRSI) chemical shift imaging (CSI)^51^ sequence, with either ^1^H or non-proton target nucleus, and with triggering available to facilitate cardiac acquisitions, as described in our previous work^15^. For prospective position update of a MRI/MRS pulse sequence, the acquisitions are preceded by a freely selectable number of ^1^H MR image navigators. The motion compensation module broadcasts the latest translation $\textbf{x}$ to the target sequence. Here, the actual prospective update is realized by updating the slice as well as the in-plane positions every TR. In SVS, the slice selective RF pulses’ carrier frequencies are recalculated to resemble the current voxel position. Similarly, this is done in slice-selective CSI excitation. All CSI phase-encoding gradients are recalculated to match the updated FoV position and the current k-space location. In short, all sequence events involving the FoV are calculated de-novo every TR.

### Phantom validation

Three phantom experiments were performed to verify correct tracking, feedback and motion update. In the first, to exclude B_1_ effects of the surface coil, the patient table was moved manually between acquisitions, displacing the phantom and the coil simultaneously. The ^31^P signal of a plastic sphere (*d* = 20 mm) filled with 1 mol/l K_2_HPO_4_/KH_2_PO_4_ was imaged using a ^31^P/^1^H surface coil (*d*(^1^H loop) = 10.5 cm, *d*(^31^P loop) = 9.5 cm, Rapid Biomedical, Rimpar, Germany) and a STEAM sequence integrated into the described framework for motion handling. One coronal image navigator was used for image-based tracking, acquisition details are listed in Suppl. Table S1 in the Supplementary Materials. The acquisition parameters of the ^31^P STEAM MRS sequence were 14.7 ms T_E_, 8.6 ms T_M_, 4 s T_R_, 16 acquisitions, 20 $\times$ 20 $\times$ 20 mm voxel size, 3000 Hz bandwidth.

In the second experiment, a previously described motion phantom^20^ was imaged with the coil mounted on its static outer part. The inner compartment was filled with 100 mmol/l KH_2_PO_4_ and was driven by a stepper motor inside the cavity of the torso-shaped, water filled phantom. This allowed for discrete and controlled motion patterns along the head-foot direction. The moving part was additionally equipped with the MPT motion tracking system (Metria Innovation, Milwaukee, USA)^7^ to validate the internal image tracking against an approved external system.

The third experiment was designed to validate the efficacy of the motion compensation module within an acquisition-weighted pulse-acquire chemical shift imaging (CSI) sequence^51^. The CSI sequence had a matrix size of 16 $\times$ 32, field of view (FoV) of 160 $\times$ 320 $\times$ 80, voxel dimensions of 10 $\times$ 10 $\times$ 80 mm, a repetition time (T_R_) of 2 s, and an acquisition time (T_*acq*_) of 11 min. The plastic sphere described above was placed within a 1 L water container, mounted on top of the ^31^P/^1^H calf coil. The stepper motor was used to induce controlled, periodic displacements of the container during the measurement. The displacement was set at increments of 4 mm per T_R_, with a maximum amplitude of 40 mm every 10 steps. To complement the experiment, a 2D simulation of motion effects on phase encoded MRSI data^35^ was conducted using identical acquisition, motion and latency parameters and phantom dimensions as applied during the experiment.

Details of the image navigators used for the respective experiments are listed in Suppl. Table S1 of the Supplementary Materials.

### Performance evaluation of tracking

To evaluate the potential of the object trackers we performed an offline comparison of eight OpenCV trackers (from version 3.2, except MOSSE and CSRT, which appeared in v3.4). For each dataset, two human operators (out of three: AS, TK, SW) manually selected the location of the target on all frames using an in-house written tool (https://pdl.perl.org). The outlines of the target were selected by placing two vertical and two horizontal lines on each frame.

To avoid individual systematic errors of human readers, the operator data were shuffled to form two pseudo operators, referred to as “OP1” and “OP2”.

An independent method not available in OpenCV, *Real-Time Image-based Tracker* (RealTITracker)^52^, was also added to the comparison due to its demonstrated performance on in-vivo MRI data.

We evaluated performance metrics for these 11 algorithms and the 66 acquired datasets as described in the following.

#### Reference

The first set of manually selected positions (“OP1”) was chosen as the reference to compare all tracking algorithms to. The remaining set of operators’ choices (“OP2”) was treated as an additional tracker in the comparison, illustrating inter-operator variability. An alternative approach could be to select the established RealTITracker algorithm as reference for comparison. Results for this approach are available in Suppl. Fig. S2 of the Supplementary Materials.

#### Precision metric

For each dataset we manually selected the initial bounding box (same box for all trackers) and performed the tracking task with all algorithms. At each time point the Euclidean distance between reference motion and the tracker motion was calculated, which is a commonly used metric for *tracking precision*^19^. The motion errors of each dataset are then sorted and plot as a cumulative density function (*precision curves*). Before pooling all datasets, the individual precision curves were normalized to 100 points to account for different number of repetitions. These plots display the fraction of an image series (y-axis) where the tracked displacement was below a certain error (x-axis). We selected the values at 5 mm and 2 px as numerical metrics for comparison of trackers, which are noted as percent values in the “Results” section.

#### Tracking failure

Where available, tracking algorithm failures were reported and the respective data points were removed from the precision data. This was decided on the fact that a reported tracking failure would permit a re-acquisition of transients and therefore should not directly count as a missed measurement.

#### Spatial robustness

A good tracker for prospective applications must not rely on finely-tuned positioning of the initial bounding box, it has to allow for variability in operator selection. We therefore analyzed the effect of initial bounding box size and position by shifting and scaling the initial bounding box, frequently termed *spatial robustness*^19^. 5 horizontal and 5 vertical shifts with step sizes of 2 pixels, and 5 scale variations of 2 pixel steps amount to 125 repeated evaluations for each tracker and dataset. A more detailed description can be found in the Supplementary Materials (see Suppl. Fig. S1).

#### Tracking speed

Tracking speed was reported automatically for all datasets and algorithms. Operator processing was timed manually.

#### Computation

The repeated tracking tasks for tracker comparison were performed offline on a PC with Intel i7-7700 CPU (4.2 Ghz) using a C++ implementation of the OpenCV code. MATLAB (MathWorks, Natick, Massachusetts, USA) was used for offline data handling, visualization and statistical analysis.

#### Datasets

The evaluation was performed on all mentioned datasets as listed in Suppl. Table S1 in the Supplementary Materials, including both, in-vivo and phantom image navigators.

#### RealTITracker

The Real-Time Image-based Tracker (RealTITracker)^52^ builds on the optical flow formulation by Horn and Schunck^53^. Additionally to the constraint of intensity conservation, this also assumes smoothness of the velocity field over the whole image. Zachiu et al. introduced a relaxed intensity conservation using an L1 data fidelity constraint to account for local, pulsating intensity changes from blood flow, often found in torso MRI^17^. The MATLAB implementation of the RealTITracker algorithm was downloaded (www.bsenneville.free.fr/RealTITracker) and used with the provided default parameters using 2D registration and the L2-L1 regularization. Motion vectors from background pixels (image magnitude below 10% of maximum) were disregarded. The motion field provided by the RealTITracker was compressed to a single displacement vector, similar as done in the MOSSE algorithm.

### In vivo validation

Exemplary in-vivo SVS and MRSI measurements were performed separately in two healthy volunteers at 7 T. A ^1^H/^31^P cardiac/liver surface coil was used to acquire ^31^P MR spectra from the cardiac muscle. The MRS sequence parameters were based on previously used protocols (STEAM: T_R_ 3 s, T_E_ 12.2 ms, T_M_ 7.3 ms, 64–256 averages, voxel size 75 ml, bandwidth 3000 Hz^50^; UTE-CSI: T_R_ 2 s, acquisition weighting, 3 averages, matrix 8$\times$16$\times$8, FoV 220 $\times$ 220 $\times$ 200 mm, voxel size 9.5 ml, bandwidth 6000 Hz^15^). The voxel positioning for MRS was aligned with the interventricular septum, a localization based on CINE MRI in cardiac orientations, including 2-chamber, 4-chamber and short-axis views. The measurements were cardiac triggered, ensuring that the acquisitions were accurately timed to occur during end-systole to optimize SNR^15^. Notably, the MRS acquisitions were conducted twice: once with motion compensation and once without, allowing for a comparative assessment of the impact of motion correction on the obtained spectroscopic data.

### Supplementary Information

Supplementary Video 1.

Supplementary Video 2.

Supplementary Information 3.

## Data Availability

The datasets generated and/or analyzed during the current study are not publicly available but are available from the corresponding author on reasonable request. Exemplary code for object tracking and feature combination is available from https://github.com/fantasma13/mri_tracking_example.
