# Supplementary material for: A modular motion compensation pipeline for prospective respiratory motion correction of multi-nuclear MR spectroscopy
Source: Sci Rep. 2024 May 11;14:10781. doi: 10.1038/s41598-024-61403-w (PMC11088657; doi:10.1038/s41598-024-61403-w)
Supplement: Supplementary file 3 — Supplementary Information 3. [file 41598_2024_61403_MOESM3_ESM.pdf]

A Modular Motion Compensation Pipeline for Prospective Respiratory Motion Correction of Multi-Nuclear MR Spectroscopy

Stefan Wampl, Tito Körner, Martin Meyerspeer, Maxim Zaitsev, Marcos Wolf, Siegfried Trattnig, Michael Wolzt, Wolfgang Bogner, Albrecht Ingo Schmid

Supplementary Materials

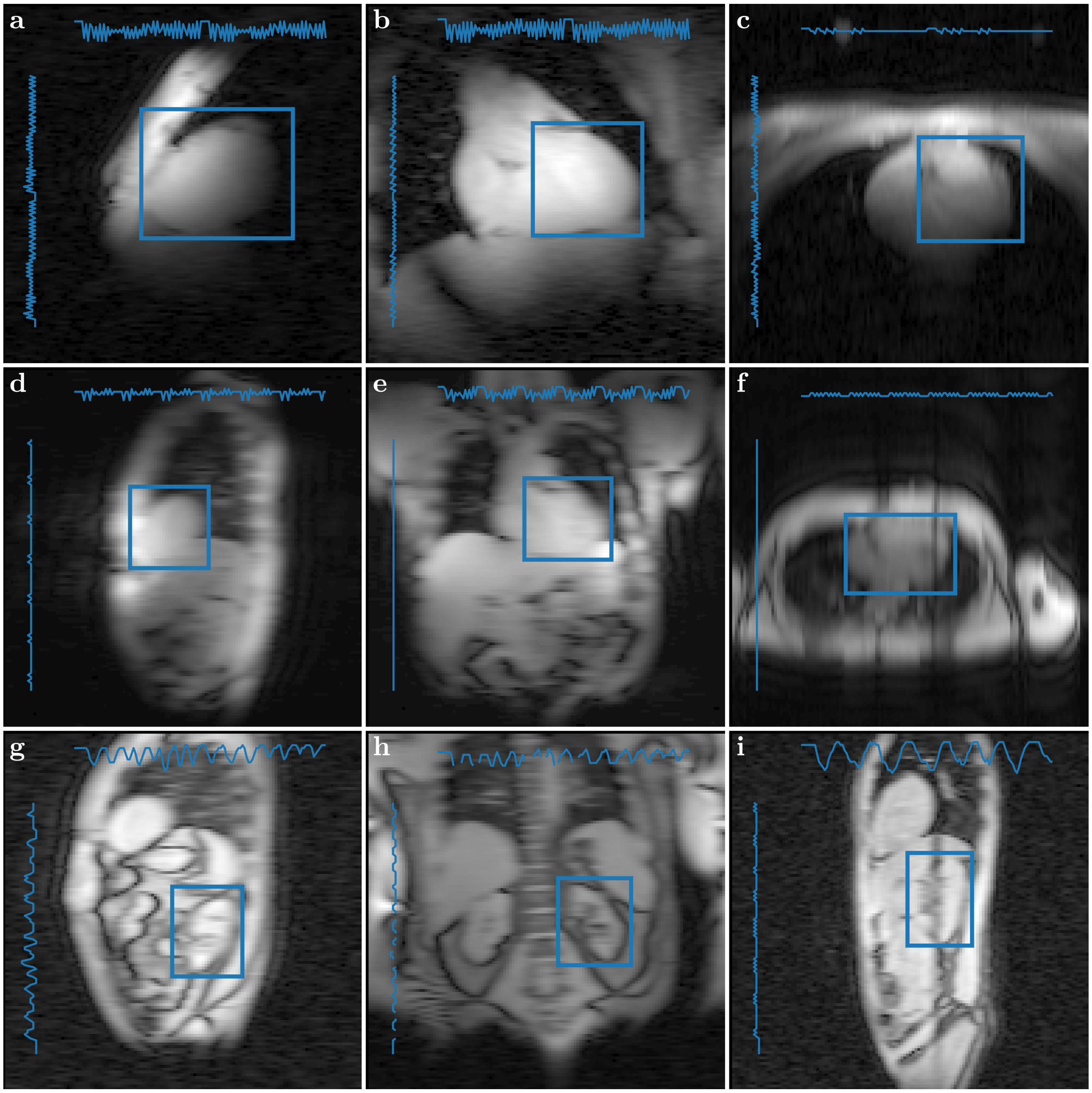

Supp. Video 1. Representative image navigators. The corresponding video (online) shows the whole time series of image navigators as presented in Figure 2 of the main manuscript. The still frame of the video is displayed above. Organ tracking is performed using the KCF tracker. Lineplots indicate the motion amplitude along x- and y-direction for the whole time series. Displacement amplitude is to scale of the respective image resolution.

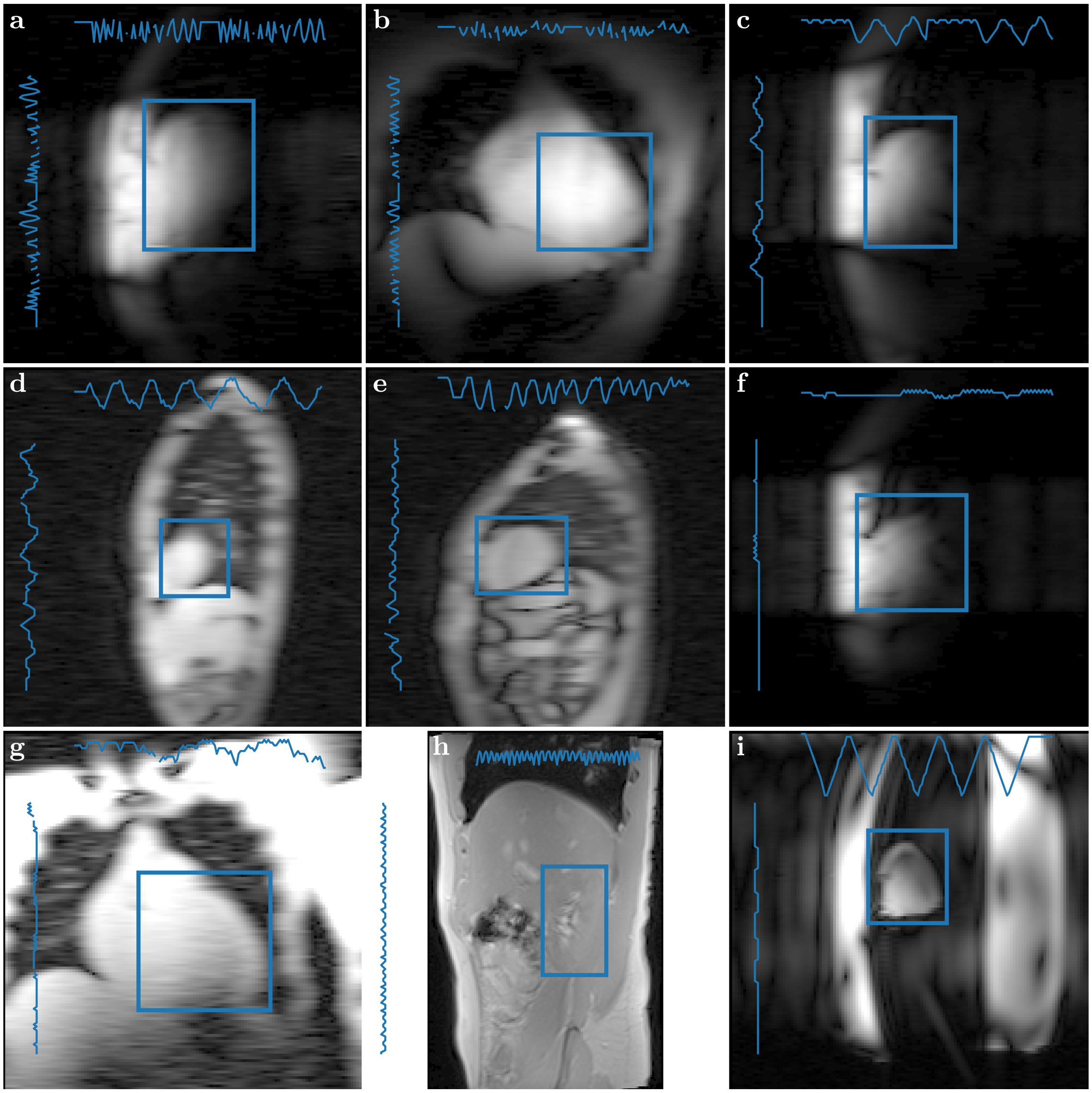

Supp. Video 2. Challenging image navigators. The still frame above shows a collection of challenging image navigator series. The corresponding video (online) features organ tracking using the KCF tracker. Lineplots indicate the in-plane displacements of the time series. Motion amplitudes are to scale of the respective image resolution. (a) and (b) show sagittal and coronal cardiac series using a single loop surface coil. The series exhibit large motion amplitude of the chestwall and consequente strong changes of coil sensitivity in the coronal slice. (c) confirms good handling of partial occlusion by the KCF tracker on a sagittal cardiac series. (d) and (e) show predominant thoracic and abdominal breathing, respectively, acquired from the heart using whole body excitation and multi-array reception. (f) presents an untriggered cardiac series with good handling of cardiac contraction. (g) illustrates noisy cardiac acquisitions with good performance of the tracker as well. (h) demonstrates the feasibility of the pipeline for post-processing using a high-resolution CINE dataset of the kidney. (i) depicts phantom acquisitions as performed for the validation.

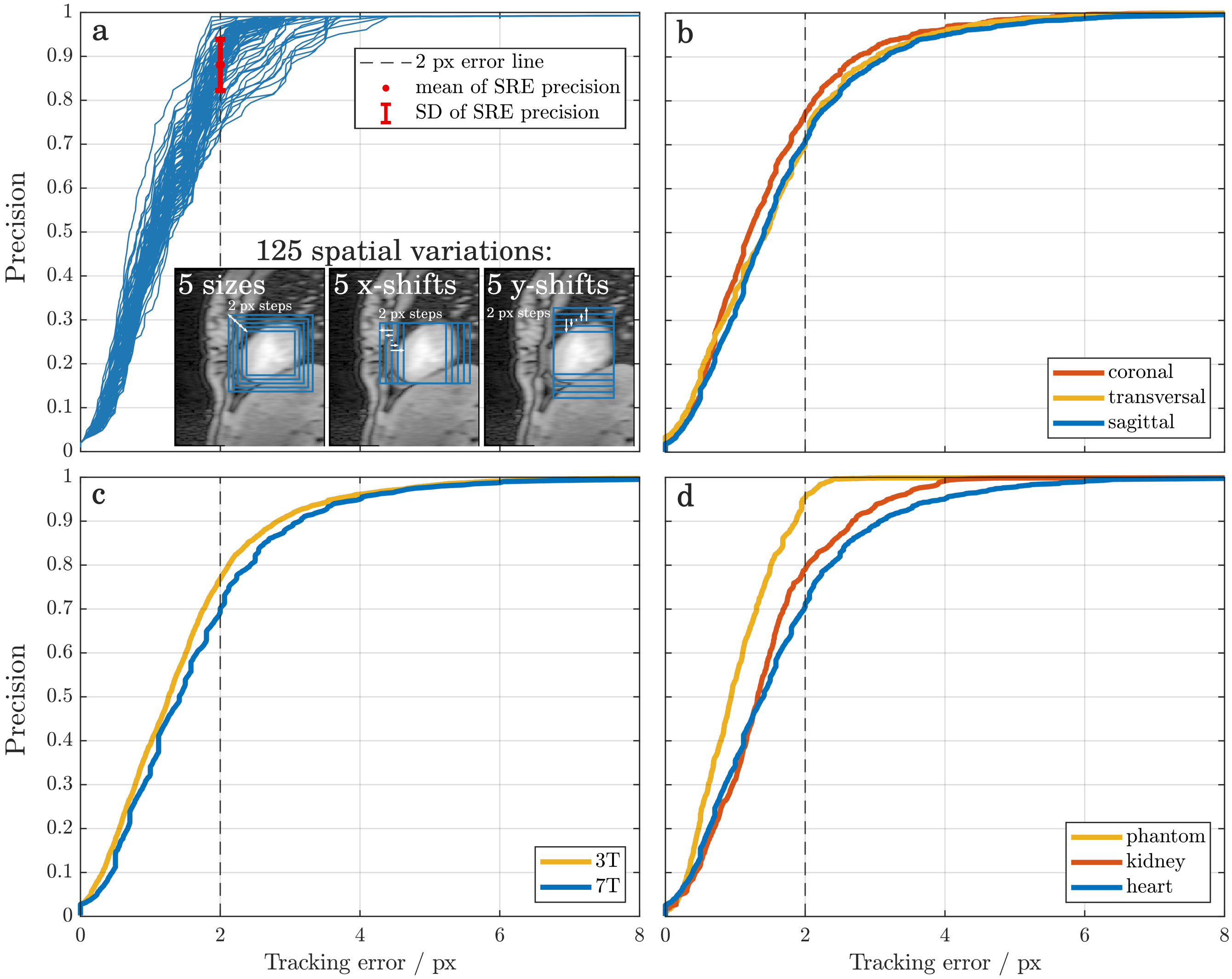

Supp. Fig. 1. Precision plots for spatial robustness evaluation (a), slice orientation (b), field strength (c) and organ comparison (d).
(a) Exemplary precision plots for the spatial robustness evaluation of a single dataset using the KCF tracker. The tracking task was repeated 125 times with different initial bounding boxes (5 horizontal and 5 vertical shifts with step sizes of 2 pixels and 5 scale variations of 2 pixel steps). The graph shows the 125 individual precision plots for a single volunteer using the KCF tracker to illustrate the idea. The mean and the standard deviation at tracking error (here: 2 px) were extracted for each dataset, as summarized for all datasets in Figure 2.
(b) Comparison of tracking performance of the KCF tracker (reference: operator) regarding the three different slice orientations sagittal ($n = 26$), coronal ($n = 23$) and transversal ($n = 17$). There is no apparent difference between sagittal and transversal slices. The tracker performs slightly better on coronal images with negligible practical effect.
(c) Tracking performance of the KCF tracker with regard to the field strength shows no substantial difference between 3 T ($n = 29$) and 7 T ($n = 37$). Slightly improved tracking on 3 T scanners can be attributed to more homogeneous B_+^1 field from the whole body coil, better spatial coverage from array coils and higher image resolution.
(d) Tracking performance of the KCF tracker with regard to the two examined organs heart ($n = 21$) and kidney ($n = 3$), and the phantom data ($n = 2$). Tracking of phantom navigators obviously performs better than in vivo data. This is mainly due to the slightly higher imaging resolution (see Table 1). Additionally, the tracked phantom was a heart-shaped object filled with phosphate containing liquid moving through air. This inherently provides a better edge contrast than the in vivo case, where organs (e.g. the heart) are surrounded by other tissues (e.g. liver, chest wall). Varying coil sensitivity at the location of the imaged organ can further influence the comparison between in vivo data and phantom. Acquisitions from the heart covered a much more diverse set of imaging parameters, as e.g. image resolution or T_R (see Table 1). The higher number of imaged volunteers with varying body compositions, breathing patterns and coil positions added to the variablity in the data. Those reasons combined resulted in a marginally worse tracking performance as compared to the kidney data.

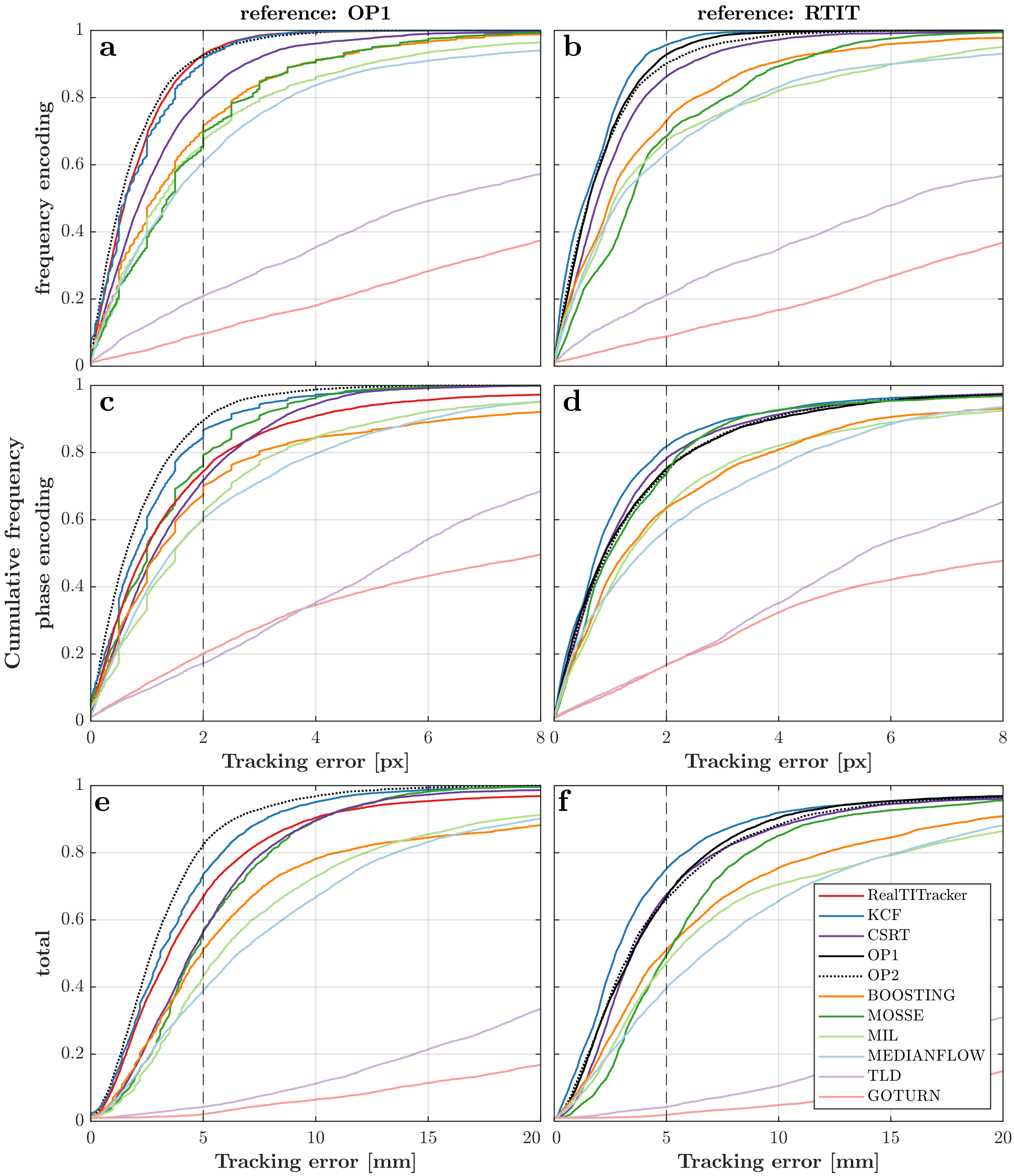

Supp. Fig. 2. To analyse influence of phase-encoding direction with lower acquisition resolution on the tracking precision, the precision curves of all datasets combined are shown for the frequency (a,b) and the phase (c,d) encoding direction separately, as well as for their final combination (e,f). Precision along the higher resolved frequency encoding direction is notably higher but still robust along phase encoding. Additionally the difference of selecting either the manual operator (a,c,e) or the RealTITracker (b,d,f) as reference is shown. In both cases the KCF tracker performs best of all automatic trackers. When comparing tracking error to RealTITracker (f), KCF even outperforms the manual operators.

Supp. Tab.1. Acquisition parameters of all in vivo and phantom image navigators.

subject	organ	B_0 [T]	coil	slices	n_{rep}	matrix size	FoV [mm x mm x mm]	T_E [ms]	T_R [ms]	t_{slc} [ms]
#1	heart	3	1	sag	100	38 x 162	169 x 200 x 5	1.19	3.65	139
#1	heart	3	1	sag	100	38 x 162	169 x 200 x 5	1.13	2.89	110
#2	heart	7	4	sag/cor	128	32 x 128	220 x 220 x 8	1.40	5.3	170
#3	heart	7	4	sag/cor/tra	64	38 x 128	250 x 250 x 8	1.30	2.50	95
#4	heart	7	4	sag/cor/tra	128	38 x 128	250 x 250 x 8	1.30	2.50	95
#5	heart	7	4	sag/cor/tra	128	38 x 128	250 x 250 x 8	1.30	2.50	95
#6	heart	7	4	sag/cor/tra	131	32 x 128	250 x 250 x 8	1.30	2.50	80
#7	heart	7	4	sag/cor/tra	128	38 x 128	250 x 250 x 8	1.30	2.50	95
#8	heart	7	4	sag/cor/tra	128	38 x 128	250 x 250 x 8	1.30	2.90	110
#9	kidney	3	2	sag	85	126 x 154	223 x 340 x 6	2.47	4.68	721
#10	heart	7	4	sag/cor	64	32 x 128	300 x 300 x 8	1.03	2.24	72
#10	heart	7	4	sag/cor/tra	64	26 x 128	300 x 300 x 8	1.03	2.24	58
#11	heart	7	4	sag/cor/tra	32	26 x 128	300 x 300 x 8	1.03	2.24	58
#12	heart	7	4	sag/cor/tra	64	26 x 128	300 x 300 x 8	1.23	2.64	69
#12	heart	3	1+2	sag/cor/tra	128	26 x 128	450 x 450 x 8	1.11	2.35	61
#12	kidney	3	1+2	sag/cor	128	32 x 128	450 x 450 x 8	1.05	2.20	70
#13	heart	3	3	sag/cor/tra	128	26 x 128	300 x 300 x 8	1.11	2.35	61
#14	heart	7	4	sag/cor/tra	64	26 x 128	300 x 300 x 8	1.03	2.24	58
#14	heart	3	1+2	sag/cor/tra	128	26 x 128	450 x 450 x 8	1.11	2.35	61
#14	kidney	3	1+2	sag/cor	128	52 x 128	450 x 450 x 8	1.05	2.20	114
#15	heart	3	1+2	sag/cor/tra	20	26 x 128	450 x 450 x 8	1.11	2.35	61
#15	heart	3	1+2	sag/cor/tra	20	26 x 128	450 x 450 x 8	1.11	2.35	61
#15	heart	3	3	sag/cor/tra	32	26 x 128	400 x 400 x 8	1.05	2.22	58
#16	heart	7	4	sag/cor/tra	128	26 x 128	350 x 350 x 8	1.03	2.24	58
phantom										
#1	-	7	5	sag	128	128 x 128	400 x 400 x 8	1.03	2.24	287
#2	-	7	4	sag/cor	128	51 x 128	350 x 350 x 8	1.03	2.24	114
#3	-	7	6	sag/cor	128	51 x 128	300 x 300 x 8	1.03	2.24	114

n_{rep}, number of repetitions. FoV, field of view. t_{slc}, acquisition time per slice. coils: 1, Siemens 32-channel spine coil *Spine 32*. 2, Siemens 18-channel flexible surface array *Body 18*. 3, Stark dual-tuned phased-array 2-channel ^{1}H/8-channel ^{31}P coil. 4, Rapid $^{31}P/^{1}H$ cardiac/liver surface coil. 5, Rapid $^{31}P/^{1}H$ liver surface coil. 6, In house-built $^{31}P/^{1}H$ coil (Goluch et al. MRM (2015), 2376-2389,73(6)).
